# Validation of a vision-related activity scale for patients with retinitis pigmentosa

**DOI:** 10.1186/s12955-020-01427-8

**Published:** 2020-06-22

**Authors:** Francisco M. Costela, Konrad Pesudovs, Michael A. Sandberg, Carol Weigel-DiFranco, Russell L. Woods

**Affiliations:** 1 Schepens Eye Research Institute, 20 Staniford St, Boston, MA 02114 USA; 2 Department of Ophthalmology, Harvard Medical School, Boston, MA USA; 3 School of Optometry and Vision Science, University of New South Wales, Sydney, Australia; 4 Massachusetts Eye and Ear, Boston, MA USA

**Keywords:** Rasch, Retinitis pigmentosa, Quality of life

## Abstract

**Purpose:**

There have been few systematic reports of vision-related activity limitations of people with retinitis pigmentosa (RP). We report a merging of data from the National Eye Institute Visual Function Questionnaire (NEI-VFQ) obtained in five previous studies. We asked whether the Vision Function Scale (VFS; Pesudovs et al., 2010) which was developed for cataract patients would apply in this new population (condition).

**Methods:**

Five hundred ninety-four individuals completed a total of 1753 questionnaires, with 209 participants providing responses over at least 4 years. Rasch analysis showed that the 15-item VFS was poorly targeted. A new instrument created by adding four driving-related items to the VFS had better targeting. As an indirect validation, VFS-plus person scores were compared to visual field area measured using a Goldmann perimeter, to the summed score for the combined 30–2 and 30/60–1 Humphrey Field Analyzer programs (HFA), to 30-Hz full-field cone electroretinogram (ERG) amplitude, and to ETDRS visual acuity. Changes in VFS-plus person scores with age and between four common heredity groups were also examined.

**Results:**

The Rasch model of responses to the 19 VFS-plus items had person and item separation of 2.66 and 24.43 respectively. The VFS-plus person scores were related to each vision measure (*p* < 0.001). Over a five-year period, there was a reduction in person scores of 0.5 logits (*p* < 0.001). Person scores fell by an average of 0.34 logits per decade (*p* < 0.0001). Participants with an X-linked hereditary pattern had, on average, lower person scores (*p* < 0.001).

**Conclusions:**

The VFS-plus instrument quantified a highly-significant annual reduction in perceived vision-related ability over a five-year period. The outcome was consistent with clinical measures of vision, and detected lower perceived vision-related ability in participants with X-linked disease. It may be of use in future studies, but this needs to be tested in a representative population sample.

## Introduction

Retinitis pigmentosa (RP) is a heterogeneous group of disorders that usually result in an initial loss of rod photoreceptors, followed by cone photoreceptors, and, possibly, inner retinal reorganization. The prevalence of RP is about 1:4000 [1], with about 100,000 affected people in the USA and two million affected worldwide. There is no approved therapy to stop disease progression or restore vision, so current management aims to slow degeneration, to treat complications, and to provide rehabilitation and psychosocial support. The primary interventions are vitamin A palmitate [2], docosahexaenoic acid (DHA) or fish oil [3, 4], and lutein [5], which, on average, slow loss of function. Potential interventions to preserve areas of vision include retinal implants, gene therapy, and stem cells. Patients with RP typically experience impaired dark adaptation and night blindness in adolescence followed by loss of mid-peripheral and then far-peripheral visual field (VF) in adulthood, resulting in tunnel vision and often blindness before the seventh decade [6–11]. Decline of VF area varies between genetic types from 2.9 to 8.1% per year [9, 12, 13], with considerable variation among patients with the same genetic type [12, 13].

Patients with RP often have difficulties with activities of daily living [14, 15]. Most patients are impaired in mobility due to difficulties with navigation, orientation [16–18], and obstacle detection [19–21]. Among different measures of visual function, VF size has been shown to be the best predictor of poor mobility in patients with RP [16, 17, 19, 20]. Objective measures of vision or functional ability may incompletely capture the patient’s subjective experiences of their deficit, hence the trend for the inclusion of patient reported outcomes in their patient management. Visual function questionnaires (VFQs) have been used to evaluate the subjective impact of RP, starting in at least the early 1990s [2, 22]. Some have compared VFQ responses to clinical measures [14, 17, 23–33] or to functional ability [27, 31, 34–41]. However, some VFQs used with RP have not been formally tested for validity.

The National Eye Institute Visual Function Questionnaire (NEI-VFQ) became available in about the year 2000 [42–44]. While there are known problems with the NEI-VFQ [45–51], including that subscales are not psychometrically sound and it is flawed by multidimensionality [48], and there are better instruments for low vision populations (e.g. Veterans Affairs Low Vision Visual Functioning Questionnaire [52, 53], Impact of Vision Impairment [54, 55]), the NEI-VFQ has been the most widely used instrument in RP samples. The NEI-VFQ measures the impact of vision impairment on emotional well-being, social functioning, and tasks related to daily vision-related activities. In studies of RP samples that have used the NEI-VFQ [4, 14, 25–37, 40, 56–58], the composite [4, 23–26, 28, 30, 32, 33, 56], sub-scale [4, 14, 27, 28, 31–34, 57, 58], or individual item [25, 40] scores have been used.

Rasch analysis [59] is a modern psychometric method that transforms raw ordinal data into an equal–interval scale using logarithmic transformations of the raw data and probabilistic equations. It provides estimates of person ability (person measure) and item difficulty (item measure) along a common measurement continuum expressed in log-odd units (logits). In the analysis used in this paper, a positive item logit indicates that the item requires a lower level of visual ability than the average (i.e., the item is relatively easier to perform). A positive logit for a participant (person ability) suggests that the participant’s visual ability is greater than the mean required level of ability for the item. In addition to item and person measures, rating scale functioning [60, 61], fit statistics, person and item separation statistics, targeting, and differential item functioning (DIF) can also be measured to assess how well the instrument works. The NEI-VFQ has been evaluated in low vision samples using modern psychometric methods such as Rasch analysis [45–47, 50, 51], showing flaws that may vary with the sample [48], but its appropriateness has not been evaluated in a RP sample.

We report an analysis merging data from five studies that created a large sample of patients with RP, many with multiple visits. We determined their vision-related activity limitations and evaluated instrument validity with comparisons to clinical visual function measures. Also, we examined changes with age and with time since first test (maximum, 5 years) and differences between some heredity categories.

## Methods

### Study population

**Fig. 1:**
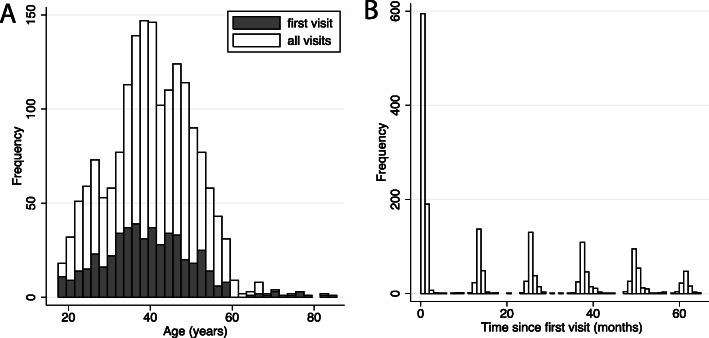
Distribution of (**a**) age of 594 individuals at first visit and all 1753 visits; and (**b**) time of visits since first visit

We used a literature search to identify studies of RP samples that used the NEI-VFQ. We used keyword “Retinitis pigmentosa” in combination with “NEI-VFQ”, “quality of life”, “activity limitation”, “activity limitations”, “VFQ-25”, or “VFQ” in Google Scholar and Pubmed from 2000 to 2020. In the 16 studies that we identified, there were a reported total of 1280 subjects with RP. Authors were contacted requesting that they share the raw data from their study, that is, the responses to each item, as well as age, gender and heredity category. If the authors were able to share data, we obtained copies of their local institutional board or equivalent documentation of approval. A flow diagram of our systematic review is included in the supplementary material. Raw data were provided for studies conducted at the Centre for Eye Research Australia (CERA, Melbourne, Vic., Australia) [62], the University of Derby (Derby, UK) [34], and at Veterans Affairs Medical Center, Salisbury, NC (VASNC) [25]. Data were also available for two studies in which some of the authors had been engaged: one study conducted at Massachusetts Eye and Ear Infirmary (MEEI), Boston, MA [3, 4, 57]; and one conducted at the Schepens Eye Research Institute (SERI), Boston, MA [40]. From those five studies, we obtained 1753 completed NEI-VFQ questionnaires from 594 individuals (aged 18 to 85 years; Fig. 1a), the majority of whom were from the MEEI study (Table 1; supplementary Table S1).

**Table 1:** Summary of the available data. The average time since the first visit is shown. The count is the number of completed questionnaires at that visit

**Study**	**Visit**	**Months**	**Count**
*CERA*	1	0	22
*Derby*	1	0	105
*MEEI*	1	0	426
*MEEI*	2	1.5	197
*MEEI*	3	14	218
*MEEI*	4	26	216
*MEEI*	5	38	215
*MEEI*	6	50	209
*MEEI*	7	61	95
*SERI*	1	0	14
*SERI*	2	7	9
*VASNC*	1	0	27
		Total forms	1753

Subject counts do not all match those reported in the original papers as some subjects did not complete the NEI-VFQ, and sometimes we obtained data for subjects who were not enrolled, did not complete the study or met requirements for inclusion in data analyses and thus were not reported in the original reports. For all five studies, the original data collection was done in person and followed a protocol approved by the local institutional review board or equivalent. The current (retrospective) study followed the tenets of the Declaration of Helsinki and was approved by the Massachusetts Eye and Ear Human Studies Committee.

Of the 594 individuals, 209 participants provided data over a period of at least 4 years and 95 provided data for 5 years (Table 1; Fig. 1b). In our sample, the inheritance pattern was known for 433 subjects, of whom 109 were dominant, 95 were recessive, 39 were X-linked, and 190 were isolated (enough family history was known to rule out recessive), while the remaining 161 were unknown or atypical RP.

Table 2 shows the characteristics of all the patients who contributed questionnaires, by study and overall. There were no significant differences in gender [Pearson χ^2^(4, *N* = 594) = 8.92, *p* = 0.06] between the five data sources. However, there was a significant difference in age [Kruskal Wallis, χ^2^ (4, *N* = 594) = 103.8, *p* < 0.001], with the CERA [62] sample being older than the SERI [40] sample (regression; t = 2.32; *p* = 0.021), which was older than the Derby sample (t = 2.59; *p* = 0.01) and VASNC [25] samples (t = 2.29; *p* = 0.02), which was older than the MEEI [4] sample (t = 4.22; *p* < 0.001). These differences can be attributed to the research questions being studied (e.g. the SERI study [40] included only subjects with late-stage “tunnel vision”).

**Table 2:** Subject characteristics by source of data and overall. Median and range of visual function measures are provided for first visit for each study, and overall visits

	CERA	Derby	MEEI	SERI	VASNC	Overall
Male	12 (55%)	69 (65%)	211 (50%)	7 (50%)	14 (52%)	313 (53%)
Median age (range) (yrs)	68 (31–85)	47 (18–83)	37.5 (18–57)	54 (43–77)	47 (29–59)	40 (18–85)
Median VA (range) (logMAR)	0.35 (−0.04–2.4)	NA	0.24 (− 0.10–1.06)	0.34 (− 0.04–0.70)	0.51 (0.10–1.58)	0.24 (−0.10–2.4)
Median Goldmann (range) (deg.^2^)	235 (35–5958)	NA	5533 (74–14,119)	222 (54–3351)	NA	6152 (35–16,679)
Median HFA (range) (dB)	NA	NA	1089 (269–3449)	74 (13–119)	NA	981 (1.2–3449)
ERG (range) (μV)	NA	NA	2.28 (0.06–62.6)	NA	NA	2.28 (0.06–62.6)

*NA* not available

### Measures of visual function

As an indirect validation of the visual-functioning person scores from the Rasch analysis (described below), we examined relationships with Goldmann perimetric area, Humphrey Field Analyzer (HFA) threshold, 30-Hz electroretinogram (ERG) amplitude, and visual acuity (VA). Availability of the visual-function measures is indicated in Table 2 which reports median and range for each metric by study and across all visits. Goldmann perimetry was measured monocularly using a V4e white stimulus in the CERA, MEEI and SERI studies, and its area was quantified using simple planimetry. As we were only interested in a first-order relationship, we did not employ Dagnelie’s solid-area correction method [63, 64]. Spearman correlation between the left and right Goldmann VF areas was 0.97. Binocular Goldmann VF was defined as the average of the two monocular VF areas. This is imperfect, as some VFs in RP are asymmetric, with wider peripheral vision temporally, which introduces a systematic under-estimation for those individuals. Median VF area in our sample population was 6152 deg.^2^ (the area of a circle with a radius of 44°) and ranged from 35 to 16,679 deg.^2^ (radii of 3.3 to 73°). The distribution of VF areas is shown in the supplement (Figure S1). Many subjects had peripheral islands of residual vision (e.g. [65]). There were significant differences in VF total area between the samples (Kruskal-Wallis, χ^2^(2, *N* = 481) = 64.1, *p* < 0.001), with the MEEI sample having larger VFs than the CERA sample (regression, t = 5.95, *p* < 0.001), which was not significantly different from the SERI sample (t = 0.28, *p* = 0.78). The effect of age and heredity group on Goldmann VF area is shown in the supplement (Figure S2).

Static perimetry was measured using a HFA with the 30–2 and 30/60–1 patterns in the MEEI study and with the 10–2 pattern in the SERI study. The SERI study targeted people with “tunnel vision”, so their VF diameters were less than 20°. All HFA tests were conducted monocularly using the size V stimulus to minimize floor effects. For MEEI data, the sensitivities of the 138 stimulus locations were summed. For SERI data, sensitivities over the four 10–2 pattern stimulus locations that corresponded to 30–2 pattern locations were summed. Binocular HFA sensitivity was defined as the average of the two monocular HFA summed sensitivities. Median summed sensitivity in our sample population was 981 dB and ranged from 1.25 to 3449 dB. The distribution of summed sensitivities is shown in the supplement (Figure S3). The MEEI sample had better VFs (higher summed sensitivities) than the SERI sample (Wilcoxon rank-sum, z = 8.21, *p* < 0.001). The effects of age and heredity group are reported in the supplement (Figure S4). For statistical analyses, we used the logarithm of the binocular HFA summed sensitivities.

ERG amplitude has been shown to be correlated with visual field size for patients with RP [66]. ERGs were monitored with a bipolar contact lens electrode on the anesthetized cornea. Cone-isolated responses to 30-Hz full-field flashes of white light (0.2 cd-sec/m^2^) were differentially amplified, tuned by a narrow bandpass filter, and summed over a period spanning about 8 min to allow amplitude stabilization in the MEEI study [4]. This process allowed amplitudes as small as 0.05 μV to be detected. Spearman correlation between the left and right ERG amplitudes was 0.91. Binocular ERG amplitude was defined as the average of the two monocular ERG amplitudes. Median ERG amplitude in our sample population was 2.28 μV and ranged from 0.065 to 62.6 μV. The distribution of ERG amplitudes is shown in the supplement (Figure S5). The effects of age and heredity group on ERG amplitude are shown in the supplement (Figure S6). For analyses, we used the logarithm of the binocular ERG amplitude, as this provided an approximately normal distribution.

Monocular VA was measured using Bailey-Lovie style charts [67] with letter-by-letter scoring [68] in four studies. Spearman correlation between the left and right eyes was 0.84. Binocular VA was defined as the VA in the better eye. Median binocular VA in our sample population was 0.24 logMAR (20/35), and ranged from − 0.10 to 2.4 logMAR (20/16 to 20/5000). The distribution of VAs is shown in the supplement (Figure S7). There were differences in VA between the samples (Kruskal-Wallis, χ^2^(3, *N* = 481) = 25.4, *p* < 0.001): VA in the MEEI sample was not better than in the SERI sample (regression, t = 0.30, *p* = 76), which was slightly better than in the CERA sample (t = 2.33, *p* = 0.02), which was not worse than in the VASNC sample (t = 1.57, *p* = 0.12); VA in the VASNC sample was worse than in the MEEI and SERI samples (t ≥ 3.79, *p* < 0.001). The effects of age and heredity group on VA are shown in the supplement (Figure S8).

### National eye institute visual function questionnaire (NEI VFQ)

The NEI VFQ [42] assesses various vision-related quality-of-life dimensions (activity limitations) using three types of rating scale categories: difficulty, frequency, and agreement. Our initial approach was to use the 15 items called the Vision Function Scale (VFS; the first 15 items in Table 3) that followed the LFVFS39 model (long-form visual functioning scale derived from NEI VFQ-39), as described in Pesudovs et al. [48] We tested the performance of the VFS scale using typical Rasch analysis metrics and found good performance (see Table S2 in the Supplement), but poor targeting, as the mean person measure was 1.85 logits (ideally, the mean person measure should be < |1| logit). To improve targeting, we investigated whether additional NEI-VFQ items could be added to the scale whilst maintaining psychometric performance. As the 15 VFS items produced a scale that was “too easy” in our sample, and since driving was known to be difficult for people with RP [69, 70], we expected that it would improve the targeting. There was a response on at least one driving item on 1538 of the 1753 completed questionnaires (least was “driving in difficult conditions” with 1225 responses).

**Table 3:** The 15 items included in the VFS (Visual Function Scale [48]) plus the additional four items that were employed in the final instrument, the VFS-plus. Original NEI VFQ-39 [42] item numbers are shown

**VFS-plus item number**	**Item description**	**NEI VFQ-39 number**
1	Eyesight	2
2	Read ordinary print in newspapers	5
3	See well up close	6
4	Find something on a crowded shelf	7
5	Read street signs or the names of stores	8
6	Going down steps, stairs, or curbs in dim light or at night	9
7	Notice objects off to the side while walking	10
8	Pick and match own clothes	12
9	Go out to see movies, plays, or sports events	14
10	Read small print in a telephone book, on a medicine bottle, or on legal forms	A3
11	Figure out whether bills received are accurate	A4
12	Doing things like shaving, styling hair, or putting on makeup	A5
13	Recognize people across a room	A6
14	Take part in active sports or other outdoor activities	A7
15	See and enjoy programs on TV	A8
16	Driving in familiar places	15
17	Driving during daytime	15c
18	Driving at night	16
19	Driving in difficult conditions	16a

### Rasch analysis

Rasch analysis produces a linear scale from categorical data (see Supplement for more detail). Our analysis was performed with Winsteps software (version 3.81) [71] according to the Andrich rating scale model for polytomous data using joint likelihood estimation. The Rasch model has one parameter for the person (ability), and one parameter corresponding to each category of an item (difficulty) [59]. Data are fit to the Rasch model (not the converse), and items that are inconsistent with the model are reviewed using item and person diagnostics. We used the outlier-sensitive fit statistic (outfit < 2.0), the inlier-pattern-sensitive fit statistic (infit < 2.0), category characteristic curves, and the average measure difference (orderly use of levels) [61]. Items that did not fit were either modified (e.g. by combining response categories) or removed [61]. To test dimensionality, we used fit statistics, graphical inspection, and principal components analysis of the residuals [72, 73]. A critical question is whether there is a coherent, unidimensional latent variable. Item separation and item reliability verify the item hierarchy, measuring the ability to stratify persons and generate reproducibility of relative item location [74, 75]. Item separation ≥3 and item reliability ≥0.9 were considered acceptable measures [74]. Person separation and person reliability verify that the instrument was able to classify person ability (e.g. distinguish between high and low ability) [74, 75]. Separation ≥2 and reliability ≥0.8 were considered acceptable measures [74]. For differential item functioning (DIF) testing, the respondents were stratified by gender, age (> 38 years and < =38 years), and studies. Significance testing for DIF is sample size dependent - we defined small or absent difference with less than 0.50 logit; minimal difference (but probably inconsequential), with difference 0.50 to 1.0 logit; and notable difference more than 1.0 logit. Testing for the appropriateness of adding or removing items incorporated all of these performance metrics. For more information on the Rasch model and its application to the NEI VFQ, see Massof et al. [76].

## Results

### Rasch analysis

The median person measure on the 15-item VFS was 1.85 (SD = 1.12) logits, indicating that the average participant’s perceived visual ability was higher than the average visual ability required by the items (conversely, average activity limitations were lower than the average item). The mean of the item measures is always automatically aligned at zero [73]. Ideally, the mean person measure should be < |1|. This poor targeting is illustrated in supplementary Figure S9. The most difficult item was item 6, “Going down steps, stairs, or curbs in dim light or at night”, which is consistent with elevated dark-adapted thresholds and mobility problems of patients with RP.

**Fig. 2:**
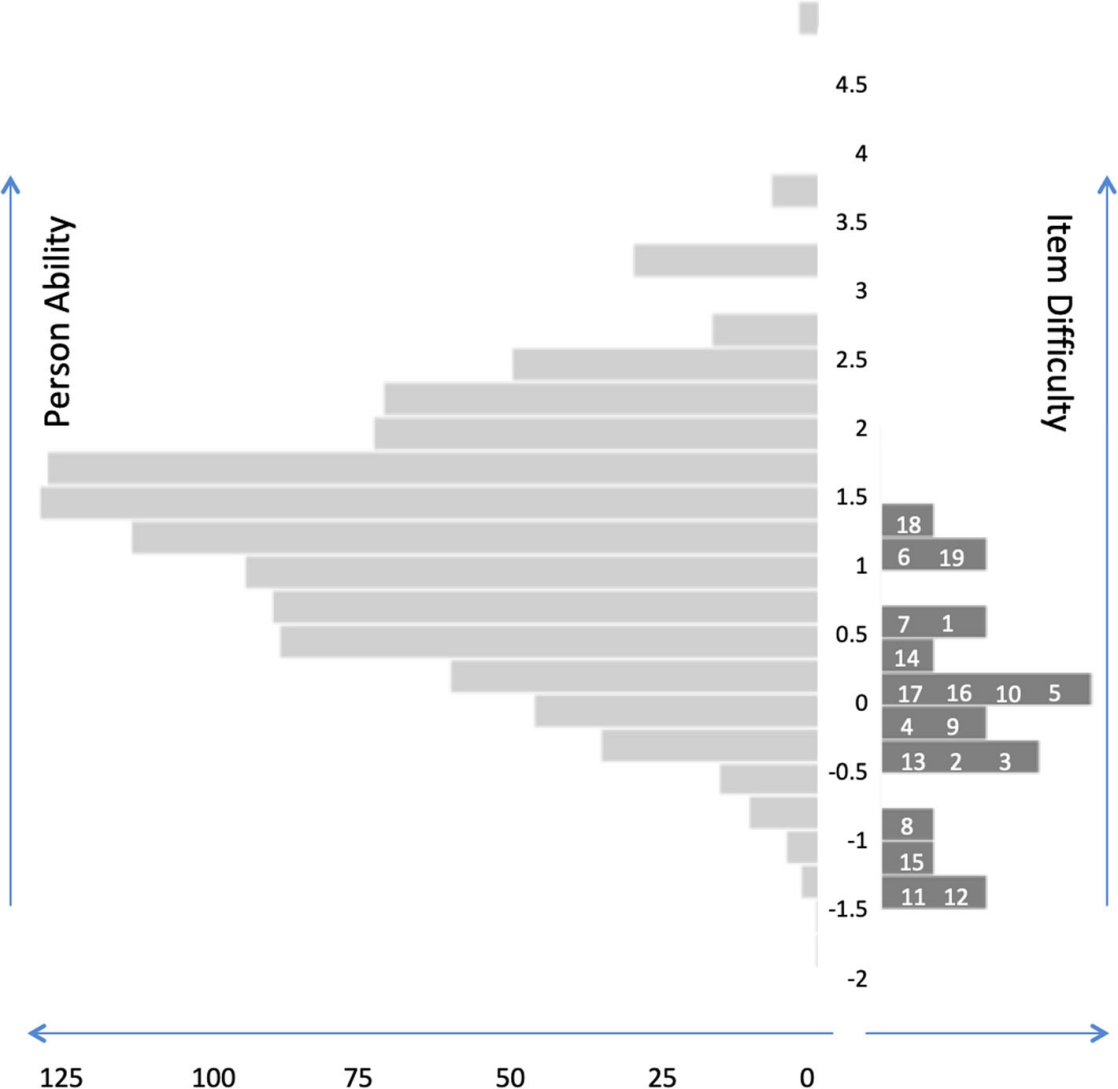
Person-item (Wright) map of the instrument. The participants are shown on the left of the vertical axis, with less able participants located at the bottom. Items (number is the item number) are located on the right of the vertical axis, with more difficult items located at the top

To improve targeting in our sample, we added the four *driving* items from the NEI-VFQ that had not been included in the VFS [48]. Targeting was improved, with the mean person measure of the 19-item instrument being 1.15 (SD = 1.10) logits (Fig. 2). We call this 19-item instrument the VFS-plus (Table 3). The most difficult items were “Driving at night” (item 18), “Going down steps, stairs, or curbs in dim light or at night” (item 6), “Driving in difficult conditions” (item 19), and “Notice objects off to the side while walking” (item 10). The easiest items were “Doing things like shaving, styling hair, or putting on makeup” (item 12), “Figure out whether bills received are accurate” (item 11), “See and enjoy programs on TV” (item 15), and “Pick and match own clothes” (item 8). With the 19-item VFS-plus, we obtained excellent fit measures, with person separation of 2.66, person reliability of 0.88, item separation of 24.43 and item reliability of 1.00 (Table 4).

**Table 4:** Overall outcomes of the Rasch analysis of the VFS-Plus instrument in our sample

Parameters	
Person	
*n* Included	1753
*n* Removed	0
Measure (logits)	1.15 ± 1.10
Infit MnSq	1.05 ± 0.64
Outfit MnSq	1.04 ± 1.01
Separation	2.66
Reliability	0.88
Item	
*n* Included	19
*n* Removed	0
Measure (logits)	0.00 ± 0.79
Infit MnSq	1.08 ± 0.47
Outfit MnSq	1.02 ± 0.47
Separation	24.43
Reliability	1
Principal component analysis (eigenvalue in first contrast)	3.3

The VFS-plus instrument was unidimensional. The average Infit and Outfit measures were around 1.0 indicating that the items, overall, fit the assumptions of the model. The most misfitting item of the instrument was item 10, “Notice objects off to the side while walking” (infit = 1.76, outfit = 1.76). That amount of misfit is usually considered acceptable. Principal component analysis of the residuals was adequate, with a variance accounting for 51.2% of the measures and the eigenvalue of the first contrast being 3.3. Differential item functioning asks whether there were differences in the responses to an item between defined groups (e.g. gender, sample source). There was no differential item functioning for age or gender. We found a few notably different responses between study groups: Items 6 and 18 were slightly harder for the CERA group, while item 8 was easier; for the SERI group, item 7 was notably harder; and for the participants in the VASNC and Derby studies, item 1 was notably easier. The rating scale performed within an acceptable range without additional adjustments (Table 5).

**Table 5:** VFQ rating scale

Category Label	Response Category	Count	OUTFIT MnSq	Andrich Threshold	Category Measure
No difficulty at all	1	1288	1.93	–	(−2.24)
A little difficulty	2	1514	0.57	−0.45	− 0.91
Moderate difficulty	3	5285	0.73	−0.98	− 0.03
Extreme difficulty	4	5646	0.73	.0.81	0.89
Stopped due to eyesight	5	14,576	1.02	.0.62	(2.20)

### Relationships with visual function

**Fig. 3:**
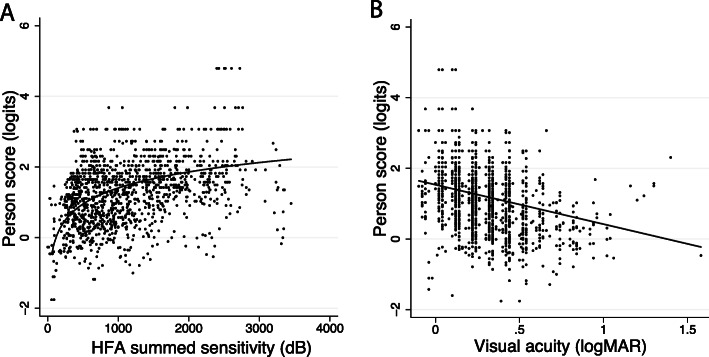
Illustration of the relationships between clinical visual function measures and person scores. Person scores increased as (**a**) binocular VF (HFA summed sensitivity) grew; and (**b**) binocular VA improved (lower logMAR)

As an indirect validation of the VFS-plus instrument, person scores were compared to the binocular visual function measures described above. We would expect that perceived ability (person score) would reduce as visual function reduced. We used mixed-effects models with subject as a random effect, which accounted for repeated measures. As was expected, instrument person scores (perceived vision-related ability) increased with increasing Goldmann VF area (z = 8.27; *p* < 0.0001), with increasing summed HFA sensitivity (z = 13.99; *p* < 0.001; Fig. 3a), with increasing full-field 30 Hz ERG amplitude (z = 10.15; *p* < 0.001), and with better VA (z = 10.63; *p* < 0.001; Fig. 3b). This is a test of concurrent validity, which supports instrument clinical validity.

### Effects of age and heredity group

**Fig. 4:**
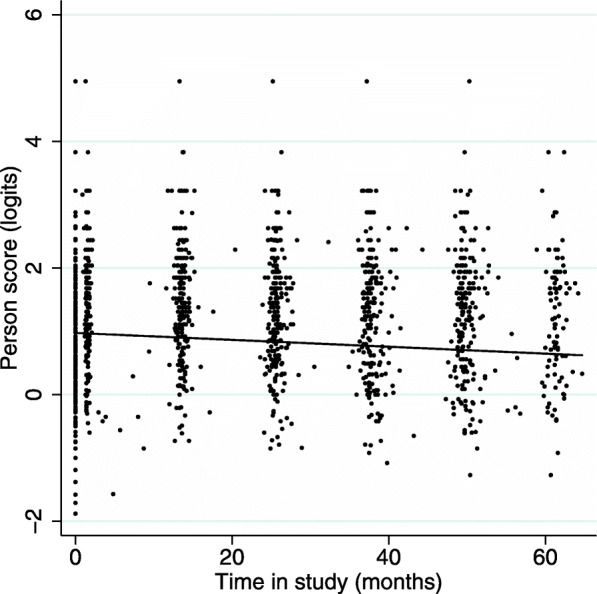
Person scores decreased over the period of the available data; maximum 65 months

We examined changes in person scores over time in study, with age and differences between heredity groups. Over the period of the available data there was a reduction in average person scores of 0.06 logits per year, from 1.16 to 0.79 logits over the 65-month period (z = 11.1; *p* < 0.001; Fig. 4).

**Fig. 5:**
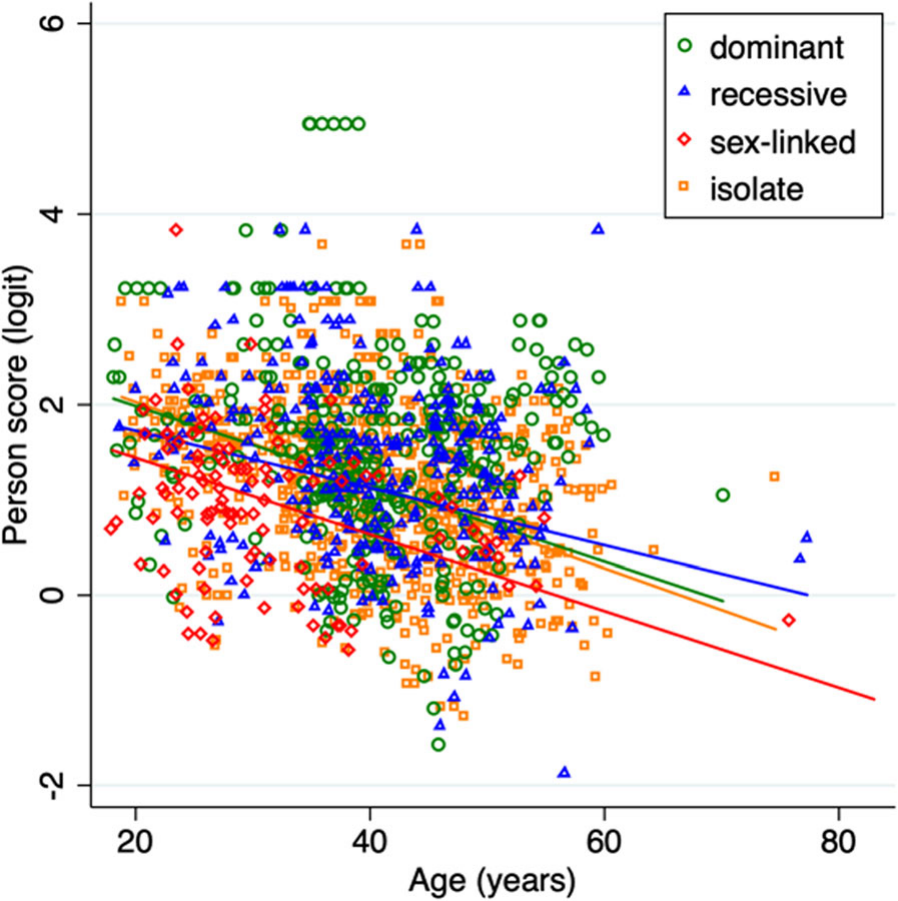
Person scores decreased with increasing age for all four common heredity groups. The person scores of participants with the X-linked hereditary pattern were lower than the other three heredity groups

Overall, person scores reduced by 0.34 logits per decade of increasing age (z = 12.18; *p* < 0.001). As shown in Fig. 5 and supplementary Figure S9, the X-linked group had lower person scores than the other heredity-pattern groups (z ≥ 2.88; *p* = 0.004). These models of the effects of age were not substantively different when subjects older than 60 years were excluded.

As a post-hoc analysis, we asked whether the visual functions were independent predictors of the person scores. In a mixed-effects model with backwards deletion of non-significant terms (*p* > 0.10), binocular summed HFA sensitivity (z = 9.05, *p* < 0.001), binocular VA (z = 7.28, *p* < 0.001), and binocular 30 Hz ERG amplitude (z = 3.54, *p* = 0.001) were all independently predictive of the person scores. Thus, those visual functions are all separately (additively) contributing to the prediction of the person scores, despite Spearman correlations (rho) between those visual function measures that ranged from 0.31 to 0.56 (*p* < 0.001).

Though the Goldmann VF area was not included in that model, that does not mean that the Goldmann VF area was not predictive. It was not included because it was strongly correlated with the HFA summed sensitivities (Spearman rho = 0.77), and was not quite as predictive of person scores as the HFA summed sensitivities. An alternative mixed-effects model that replaced the HFA summed sensitivities with the Goldmann VF area was not as good (AIC = 2048 versus 1983). In that model, Goldmann VF area was independently associated with person score (z = 3.62, *p* < 0.001), when corrected for VA and ERG amplitude.

## Discussion

Our retrospective study used data from five studies of patients with RP, many with multiple visits, who had completed the NEI-VFQ [42]. We developed and verified a modified version of the VFS (Vision Function Scale [48]) that included an additional four items (total 19 items). The poor targeting of the (15-item) VFS instrument in this sample was improved by adding the four driving-related items from the NEI-VFQ-39. Rasch analysis showed that this VFS-plus instrument provided a valid scale to determine vision-related activity limitations. Moreover, this new instrument was able to measure a reduction in perceived vision-related ability (increased activity limitations) over a five-year period and a reduction with increasing age that was consistent with expectations. Similarly, the reductions in perceived vision-related ability were related to worsening visual fields, worsening ERG amplitude, and worsening VA, consistent with expectations, as reductions in visual function would be expected to reduce the ability to perform daily activities as evaluated by this instrument, providing an indirect confirmation of instrument validity.

Consistent with the difficulties reported by people with RP [69, 70], the hardest items were 18 “Driving at night”, 6 “Going down steps, stairs, or curbs in dim light or at night”, 19 “Driving in difficult conditions”, and 7 “Notice objects off to the side while walking”. That these activities are difficult probably derives from the impaired dark adaptation, nyctalopia (night blindness), and visual field loss found in RP. Not surprisingly, the easiest items were 11 “Figure out whether bills received are accurate”, 12 “Doing things like shaving, styling hair, or putting on makeup”, 15 “See and enjoy programs on TV”, and 8 “Pick and match own clothes”, all relatively easy daily activities for persons with better preserved central vision. That these items have been ordered by the Rasch analysis as would be expected, provides confidence in the face validity of the VFS-plus instrument.

Previous studies have shown that patients with X-linked RP due to mutations in the retinitis pigmentosa GTPase regulator (RPGR) gene have faster rates of change for VA than patients with dominant RP due to mutations in the rhodopsin (RHO) gene or than patients with autosomal recessive USH2A gene (Usher syndrome type II) [77]. Legal blindness was reached at an earlier age in patients with RPGR mutations (median 45 years) than in patients with USH2A mutations (median 58 years) or RHO mutations (median 77 years) [77]. Consistent with those findings of worse visual functioning in people with X-Linked RP, we found that participants with a X-linked hereditary pattern had significantly lower person scores (Fig. 5). Previous research on rates of visual function decline [12] suggests that the reduction in VA with age may be the major factor in the worse perceptions of vision-related ability among patients with X-linked RP. In our sample, we found that VA (z ≥ 2.30, *p* ≤ 0.02), HFA summed sensitivities (z ≥ 5.15, *p* < 0.001) and 30-Hz ERG amplitudes (z ≥ 2.61, *p* ≤ 0.009) were lower in the X-linked group (see supplement). As these visual functions were associated with person scores (independent predictors), it is not surprising that the X-linked group had worse activity limitations.

Though our sample was large (594 subjects, 1753 questionnaires), it was drawn primarily (72% subjects, 90% questionnaires) from the MEEI study, a randomized controlled trial [3, 4] that had inclusion criteria designed for the purposes of that study, thus, the enrolled sample was not representative of all people with RP. Since the MEEI study sought to find drug and nutrient-related treatments for RP, it was presumed that people with late-stage RP would not benefit from treatment and thus their involvement would be futile. However, we obtained data from the majority of the people who were screened but not enrolled (*n* = 205). That full sample was more representative as it included subjects with small VFs (e.g. as shown in Table 2, Goldmann visual fields ranged from 74 to 14,119 degrees^2^). The CERA, Derby and VASNC studies were diverse samples, and the SERI sample was only subjects with tunnel vision (a group that was excluded from enrollment in the MEEI study). As shown in Table 2, the CERA, Derby and SERI studies included subjects who were considerably older than the MEEI study. The samples were moderately geographically diverse, with the MEEI study sampling widely across the USA, despite being conducted in Boston, MA, USA, the Derby study was conducted in England, and the CERA sample was obtained in Melbourne, Australia. Even so, all of those studies were convenience samples, and it is possible that people with RP who do not participate in research studies would have different responses to the items of the VFS-plus test.

In summary, we found that we could measure the increased activity limitations (reduced perceived ability) related to vision loss in a large sample of patients with RP, and relationships were consistent with expectations. The validity of the 19-item VFS-plus instrument represents a specific performance of this RP population, which did not occur in the cataract population in which the VFS was developed [48]. This VFS-plus instrument may be of use in future studies, but this needs to be tested in specific populations. Indeed, item banking studies with larger numbers of items have shown that in conditions with loss of peripheral vision (glaucoma and hereditary retinal degenerations including RP), driving items form a separate measurement scale to other activity limitation items [78, 79].

While it has not been so tested, it suggests that the 19 items of the VFS-plus instrument, could be presented on their own in future studies, which would shorten the time required to conduct the measurements and, perhaps, even allow certain clinical trials to be conducted remotely.

## Conclusions

Our instrument, based on the Vision Function Scale (from the NEI-VFQ) with additional driving-related questions, provided a valid scale tested in an RP population to quantify an annual reduction in perceived vision-related ability over a five-year period. The outcome was consistent with reductions in clinical measures and differed between some hereditary-pattern groups.

## Supplementary information

**Additional file 1.**

## Data Availability

The datasets generated during and/or analysed during the current study are available from the corresponding author on request, following demonstration of approval for handling of such data by their institutional review board or equivalent, and completion of a data use agreement.

## Abbreviations

RP: Retinitis pigmentosa
DHA: Docosahexaenoic acid
VF: Visual field
VFS: Vision Function Scale
VFQs: Visual function questionnaires
NEI-VFQ: National Eye Institute Visual Function Questionnaire
DIF: Differential item functioning
MEEI: Massachusetts Eye and Ear Infirmary
SERI: Schepens Eye Research Institute
CERA: The Centre for Eye Research Australia
VASNC: The Veterans Affairs Medical Center, Salisbury, NC
HFA: Humphrey Field Analyzer
ERG: Electroretinogram
VA: Visual acuity
