# Supplementary material for: Validation of a vision-related activity scale for patients with retinitis pigmentosa
Source: Health Qual Life Outcomes. 2020 Jun 22;18:196. doi: 10.1186/s12955-020-01427-8 (PMC7310073; doi:10.1186/s12955-020-01427-8)
Supplement: Supplementary file 1 — Additional file 1. [file 12955_2020_1427_MOESM1_ESM.docx]

# supplementary material

Costela FM, Pesudovs K, Sandberg MA, Weigel-DiFranco C, Woods RL. Validation of a vision-related activity scale for patients with retinitis pigmentosa. *Health and Quality of Life Outcomes*

Table S1. Demographics of the patients reported in our manuscript by study.

| Study | N reported | N used | Year | Status |
| --- | --- | --- | --- | --- |
| CERA (Australia) [16] | 40 | 22 | 2016 | Data included |
| Derby (UK) [21] | 105 | 105 | 2018 | Data included |
| MEEI (USA) [17, 18] | 456 | 426 | 2004 | Data included |
| Schepens (USA) [19] | 12 | 14 | 2010 | Data included |
| VASNC (USA) [22] | 27 | 27 | 2005 | Data included |
| Emory (USA) [11] | 143 | - | 2017 | Offered data but not yet provided |
| Tuebingen (Germany) [12] | 25 | - | 2016 | Offered data but not yet provided |
| Paris (France) [13] [14] | 60 | - | 2015 | Unable to provide raw data |
| Osaka (Japan) [15, 20] | 109 | - | 2018 | Unable to provide raw data |
| Seoul (Korea) [1, 3] | 144 | - | 2009 | Other reasons |
| Umea (Sweden) [23, 24] | 49 | - | 2010 | Other reasons |
| Chiba (Japan) [5, 25] | 40 | - | 2010 | Other reasons |
| Dundee (UK) [10] | 18 | - | 2010 | Other reasons |
| Aachen (Germany) [9] | 6 | - | 2012 | Other reasons |
| Granada (Spain) [2] | 26 | - | 2013 | Other reasons |
| Sao Paulo (Brazil) [26] | 20 | - | 2015 | Other reasons |


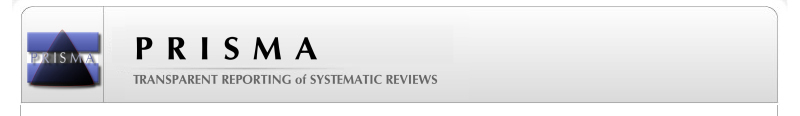
**PRISMA Flow Diagram**

Authors unable to provide the raw data (n=2)

[13-15]

Other reasons
(n = 7)

[1-10]

Studies identified through online searches in google scholar with keywords ‘NEI-VFQ’ and “retinitis pigmentosa”, etc.
(n = 13)

[1-15, 20-22]

Offered data but not yet provided (n=2)

[11, 12]

Studies identified through known collaborators

(n = 1)

[16]

Studies included in meta-analysis
(n = 5)

Centre for Eye Research Australia (CERA) [16]

Massachusetts Eye and Ear Infirmary (MEEI) [17, 18]

Schepens Eye Research Institute (SERI) [19]

University of Derby (Derby) [21]

Veterans Affairs Medical Center, Salisbury, NC (VASNC) [22]

## Acquisition

## Included

## Identification

Total studies identified with reported records of RP-NEIVFQ questionnaires from population with retinitis pigmentosa

(n = 16)

Studies identified through co-authors directly involved in those studies
(n = 2)

[17-19]

| 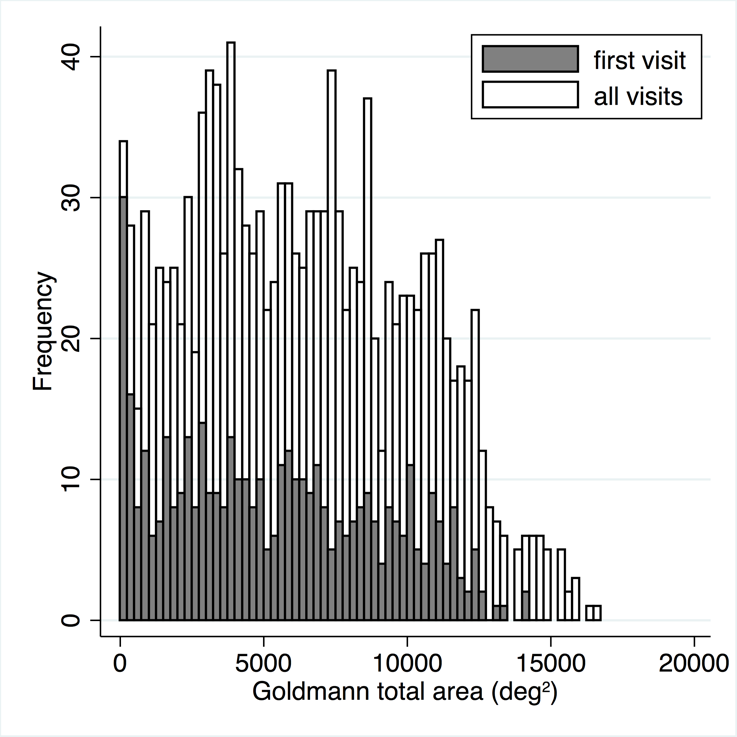 |
| --- |
| Figure S1. Distribution of binocular total visual field (VF) area measured using a Goldmann perimeter of all subjects at first visit and all visits. |

| 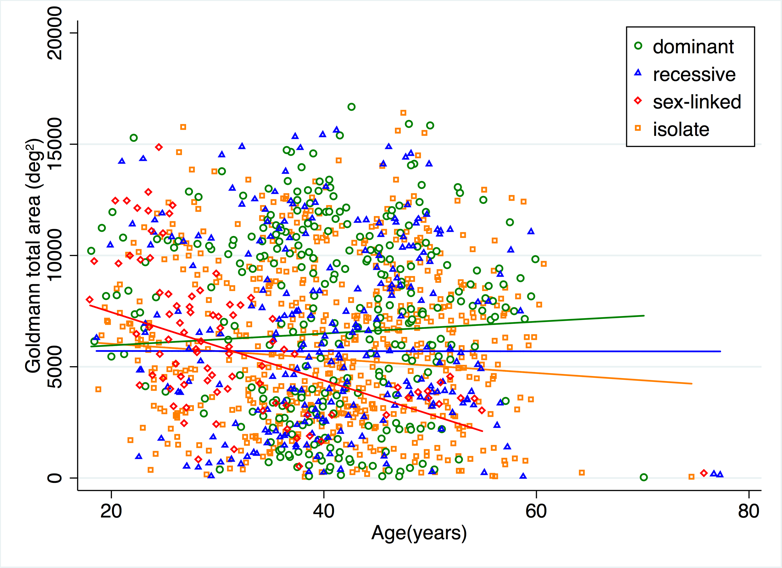 |
| --- |
| Figure S2. Binocular VF area of subjects with the X-linked hereditary pattern decreased with age (z=2.62, p=0.009), but the other heredity groups did not (z≤0.78, p≥0.43). |

**Binocular HFA (Humphrey Field Analyzer) summed sensitivities**

|  |
| --- |
| Figure S3. Distribution of binocular HFA summed sensitivities of all subjects at first visit and all visits. |

| 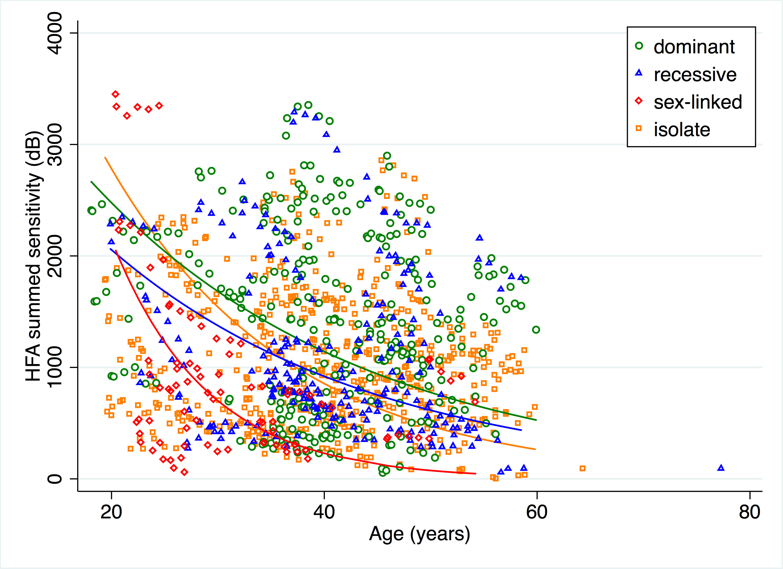 |
| --- |
| Figure S4. Binocular HFA summed sensitivities decreased with age, and the X-linked group decreased more quickly with age (worse VF; z=10.84, p<0.001) than the other heredity groups (z≥4.77, p≤0.001). The X-linked group, also had worse VFs than the other heredity groups at a given age (z≥5.15, p≤0.001). The fits are curved as the data was modeled with ln(summed sensitivity). |

**Binocular ERG (electro-retinogram) amplitudes with 30Hz stimulus**

|  |
| --- |
| Figure S5. Distribution of binocular ERG amplitudes with 30Hz stimulus of all subjects at first visit and all visits. |

| 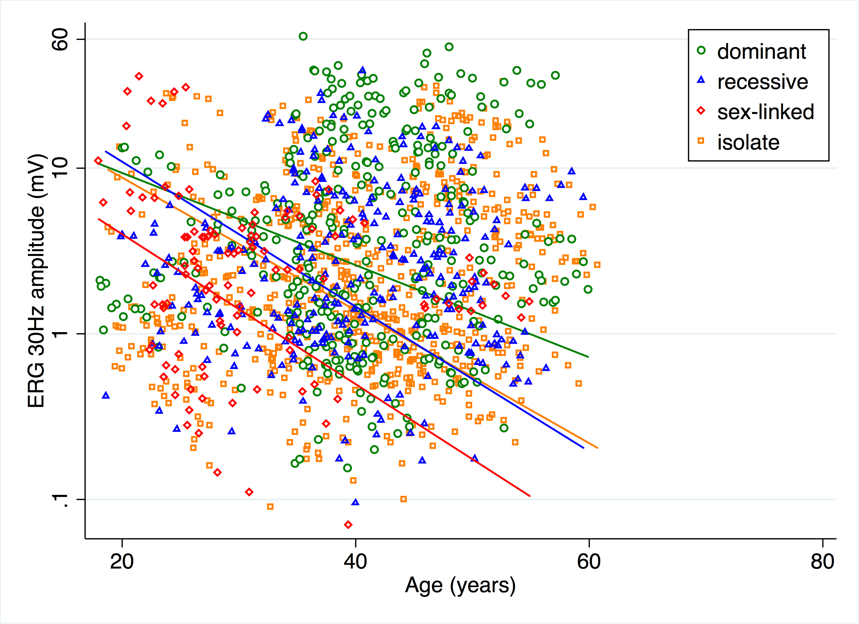 |
| --- |
| Figure S6. Binocular ERG amplitudes with 30Hz stimulus decreased with age, and the X-linked group had lower amplitudes at a given age than the other the other heredity groups (z≥2.61, p≤0.009). The ERG amplitudes decreased more slowly in the dominant group than the recessive (z=2.88, p=0.004), isolate (z=2.69, p=0.007) and X-linked (z=2.23, p=0.03) groups. |

**Binocular VA (visual acuity)**

|  |
| --- |
| Figure S7. Distribution of binocular VA of all subjects at first visit and all visits. |

| 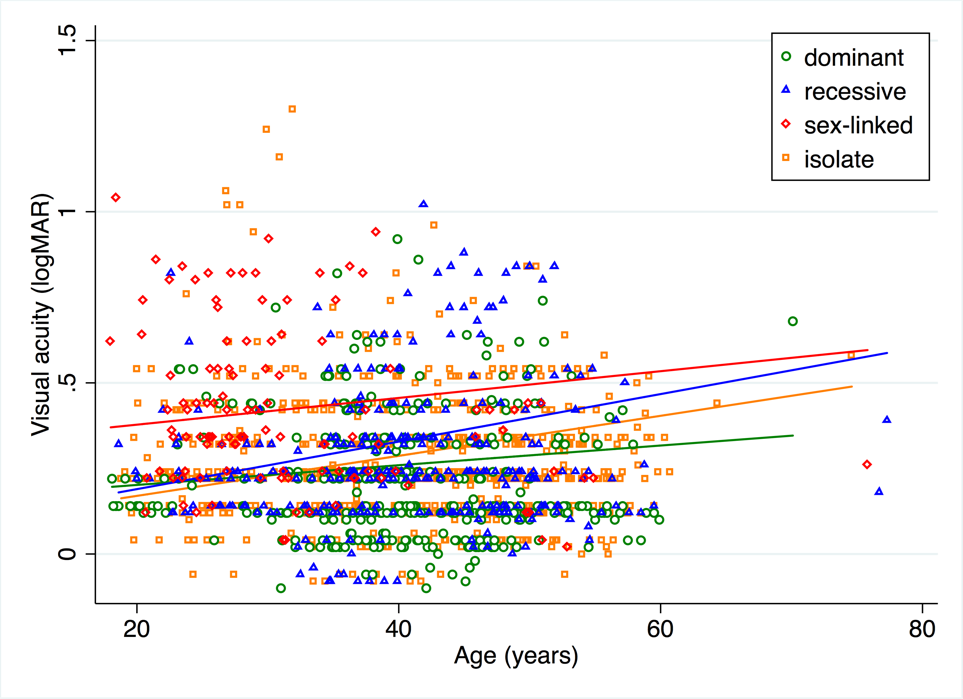 |
| --- |
| Figure S8. Overall, binocular VA worsened with increasing age (higher logMAR). The X-linked group had worse VA than the dominant (z=3.62, p<0.001), isolate (z=3.25, p=0.001) and recessive (z=2.30, p=0.02) groups. |

| 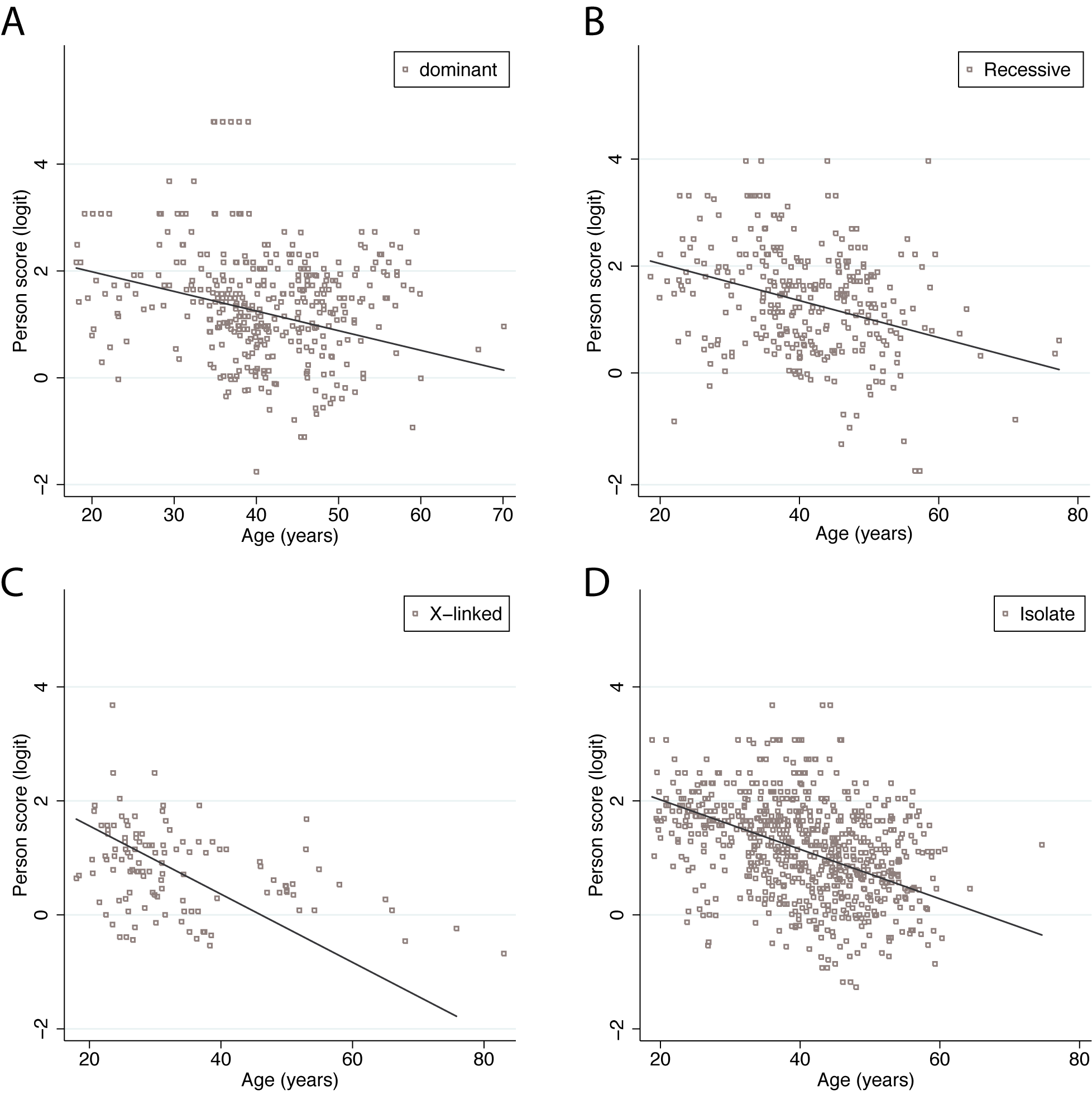 |
| --- |
| Figure S9. Person scores change with age for all four hereditary groups. |

**Rasch analysis using 15-item VFS (Vision Function Scale)** **[27]**

Rasch analysis with the 15-items of the VFS [27] was conducted using data from 489 subjects who completed 1,648 completed questionnaires that were available at the time (before obtaining the Derby data). Details of that Rasch analysis are shown in Table S2 and Figure S10 shows the Wright (person-item) map. The mean person measure on the VFS was 1.85 (SD = 1.12) logits. Ideally, the mean person measure should be < |1| logits. The hardest item was item 6, “Going down steps, stairs, or curbs in dim light or at night”, which is consistent with the dark adaptation and pedestrian mobility problems of patients with RP. Adding four driving-related, items from the NEI-VFQ to the VFS, which we called the (19-item) VFS-plus, improved the targeting of the instrument (see Figure 2 and Table 4 in main text).

Table S2 Overall outcomes of the Rasch analysis of the VFS-Plus instrument in our sample.

| Parameters |  |
| --- | --- |
| Person |  |
| *n* Included | 1,648 |
| *n* Removed | 0 |
| Measure (logits) | 1.85 ± 1.12 |
| Infit MnSq | 0.98 ± 0.73 |
| Outfit MnSq | 0.94 ± 1 |
| Separation | 2.23 |
| Reliability | 0.83 |
|  |  |
| Item |  |
| *n* Included | 15 |
| *n* Removed | 0 |
| Measure (logits) | 0.00 ± 0.98 |
| Infit MnSq | 1.07 ± 0.43 |
| Outfit MnSq | 0.92 ± 0.39 |
| Separation | 23.42 |
| Reliability | 1 |
|  |  |
| Principal component analysis (eigenvalue in first contrast) | 3.0 |

| 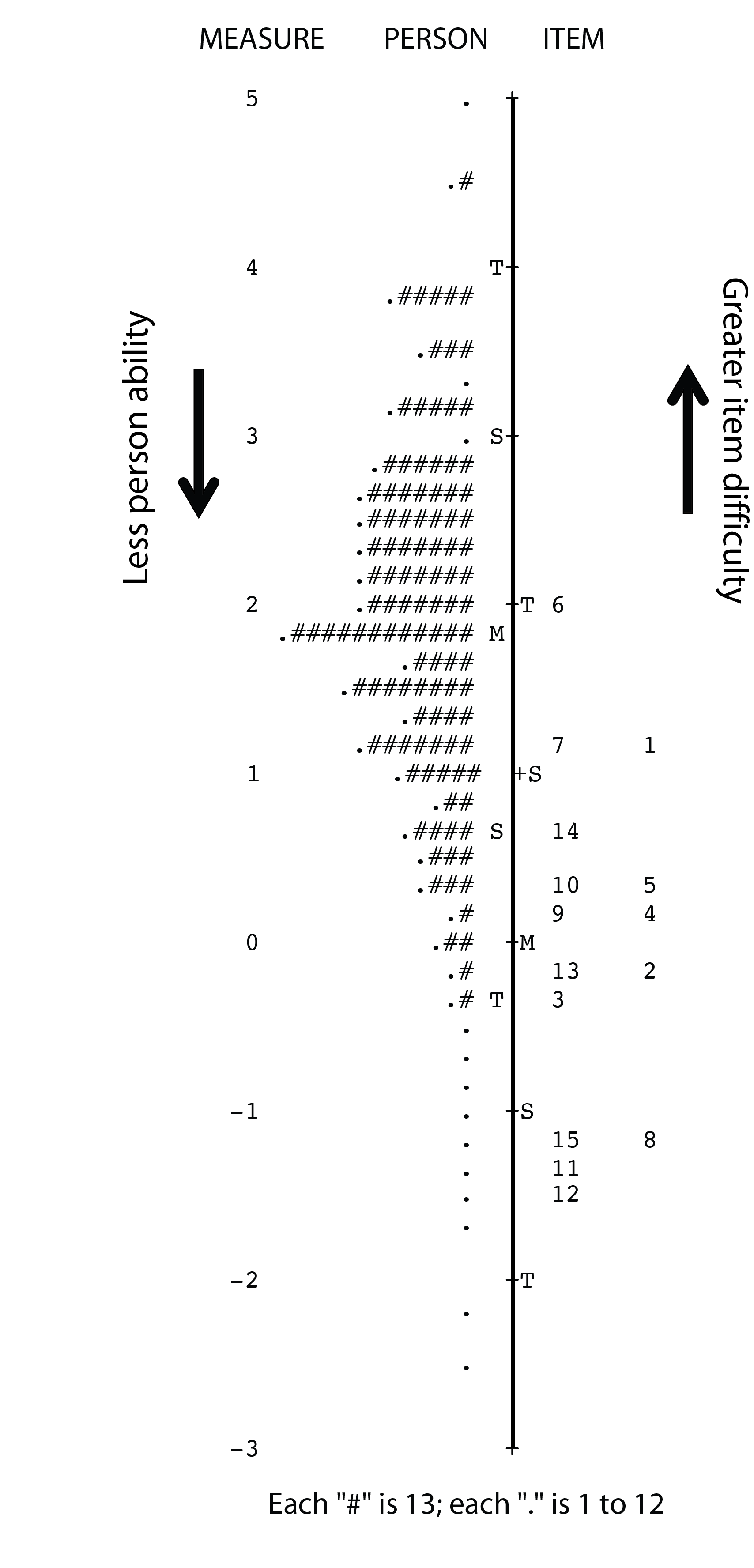 |
| --- |
| Figure S10. Person-item (Wright) map of (15-item) VFS. The participants are on the left of the dashed vertical line, with less able participants located at the bottom of the map. Items are located on the right of the dashed line, with more difficult items located at the top of the map. |

### **Rasch Analysis**

The Rasch model (invented by Georg Rasch [28]), creates measurements from categorical data, such as answers to quality of life questionnaires, as a function of the relationship between the respondent's abilities (or attitudes or personality traits) and the item difficulty. Rasch analysis is a psychometric model that is a special case of item response theory that provides a method to measure an underlying latent trait, a construct that describes an aspect of the respondent’s abilities, attitudes, or personality traits. That latent trait is inferred from the measured responses. The Rasch model provides a hierarchical order of each item based on its difficulty and an ability score for each respondent (patients in our study). It resembles a ruler, only instead of cm or inches, the scale is measured with “items.” Less difficult items are located on the bottom, more difficult items are located on the top. People can also be placed on the same measurement scale, with less able people on the bottom and more able people on the top. See figure S11 for a schematic representation of the scale with some of the items of our instrument. The model also assumes that the probability of a given respondent’s affirming the difficulty of an item is modeled by a logistic function on a linear scale of the relative distance between the item’s location and the respondent’s location. A probabilistic form of Guttman scaling [29] and a variety of statistics are used to assess the fit of the data to the model [30].

| 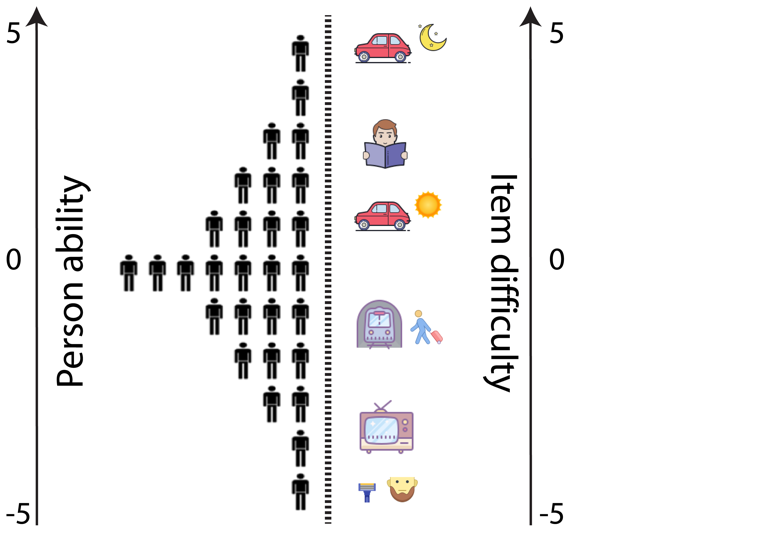 |
| --- |
| Figure S11. Schematic Rasch scaling of person ability and item difficulty along an equal interval scale. |

The model is created from actual data, the proportion of responses of each person to each test item. Thus, the scale is only dependent on the test item’s difficulty and the person’s ability. The Rasch linear measures are originally expressed in log-odd units. More specifically, scores are measured in logits (the unit of measure used by Rasch for calibrating items and measuring persons). The person-ability score is based on each individual’s performance; the more difficult the items that the patient can perform well, the higher is their ability score and vice versa. Thus, patients with worse ability show a lower person-ability score compared to those with less severe impairment. If an item is positive, it requires a higher level of activity than the mean of the items, whereas a negative item logit suggests that the item requires a lower level of activity than the average. Similarly, positive and negative person-logit score suggests that the person’s level of activity is higher and lower than the mean required level of difficulty for the items, respectively. A more thorough description of logits can be found in Ludlow & Haley [31].

When there is a lack of the expected probabilistic relationship between items (item misfit) in the scale, noise is introduced into the measurement, diminishing the instrument’s quality. There are different strategies to improve the scale in this case [32]. Items that do not fit (the model, and thus the underlying trait) or are not well-behaved either are modified (e.g. combining levels) or removed [32]. These items may be poorly constructed or measuring a different dimension (or trait). INFIT and OUTFIT statistics are the most widely used diagnostic Rasch fit statistics. Comparison is with an estimated value that is near to or far from the expected value. INFIT is more diagnostic when item measures are close to the person measures. OUTFIT is more diagnostic when item measures are far from the person measures. In the analysis described in our manuscript, we used thresholds within the outlier-sensitive fit statistic (outfit < 2.0) and the inlier-pattern-sensitive fit statistic (infit < 2.0) [32].

It is also necessary to identify whether thresholds are disordered. Disordered thresholds occur when there are too many response options, or when the labeling of options is similar to one another, potentially confusing or open to misinterpretation (e.g., not at all, hardly at all, and a little). A way of improving disordered thresholds consists in collapsing these categories, which usually improve overall fit to the model. For example, categories that are less likely to be chosen (or not appropriately used across the whole scale), can be collapsed with adjacent categories.

Once disordered thresholds are removed, fit of data to the Rasch model is assessed by examining deviations from model expectations, including DIF (differential item functioning). DIF happens when different groups within the sample respond in a different manner to an individual item, which can be detected both graphically (inspection of the item characteristic curves), and statistically (analysis of variance across each level of the person factor and levels of trait). In the analysis that we carried out in the manuscript, we stratified the respondents by gender, age, and studies.

Further, it is also crucial to confirm unidimensionality. Rasch analysis uses fit statistics, graphical inspection, and principal components analysis (PCA) of residuals to indicate whether a set of items comprises a uni-dimensional measure [33, 34]. Unidimensionality is important because it provides further evidence that the instrument is measuring the underlying, coherent, latent variable and is formally tested by allowing the pattern of factor loadings on the first residual to determine subsets of items. The first factor in the PCA of residuals should exceed 50% and the first contrast of residuals should be < 2.5 eigenvalues. Otherwise, the items are divided into separate models, constructing a new instrument for each dimension.

Next, it is necessary to examine individual item and person levels produced by the Rasch model and confirm that the scale is appropriately targeted to the population being assessed. Measurement is better when the middle values of persons lie close to the middle values of the measured items (which would indicate an excellent targeting). There are additional metrics to evaluate targeting - Item separation and item reliability verify this item hierarchy, measuring the ability to stratify persons and generate reproducibility of relative item location[35, 36]. Item separation ≥ 3 and item reliability ≥ 0.9 are considered acceptable measures [37]. Person separation and person reliability verify that the instrument is able to classify person ability (e.g. distinguish between high and low ability)[36, 37]. Separation ≥ 2 and reliability ≥ 0.8 are considered acceptable measures [37].

In summary, final measures are built by the operator based upon the best judgments of spread of item values, reduced error of measurement (precision), probability and improbability (fit) of item and person values to that expected from the model, overall reliability (noise), simplicity, and conformity to the nature of the clinical values that are being measured. Importantly and different to item response theory approaches, building measures using Rasch Analysis requires that the data fit the model, not that the model fits the data.

Rasch analysis, or the resultant instrument (that measures the trait), can detect changes in the person-ability scores following rehabilitation or intervention. An instrument needs sufficient items to provide precise measurement across the population [27, 38]. As the Rasch scoring transformation improves measurement precision through noise reduction, it leads to instruments with superior psychometric properties [39]. Optimization of scale properties can have a profound impact on measurement precision [40], for example, halving the required sample size using a Rasch-optimized instrument compared to a summary-scored instrument [41].

For more information on the Rasch model and its application to the NEI VFQ, see Massof et al. [42].

**References**

1. Seo JH, Yu HG, Lee BJ: **Assessment of functional vision score and vision-specific quality of life in individuals with retinitis pigmentosa.** *Korean Journal of Ophthalmology* 2009, **23:**164-168.

2. Chacon-Lopez H, Pelayo FJ, Lopez-Justicia MD, Morillas CA, Urena R, Chacon-Medina A, Pino B: **Visual training and emotional state of people with retinitis pigmentosa.** *Journal of Rehabilitation Research and Development* 2013, **50:**1157-1168.

3. Hahm BJ, Shin YW, Shim EJ, Jeon HJ, Seo JM, Chung H, Yu HG: **Depression and the vision-related quality of life in patients with retinitis pigmentosa.** *British Journal of Ophthalmology* 2008, **92:**650-654.

4. Sugawara T, Hagiwara A, Hiramatsu A, Ogata K, Mitamura Y, Yamamoto S: **Relationship between peripheral visual field loss and vision-related quality of life in patients with retinitis pigmentosa.** *Eye (Lond)* 2010, **24:**535-539.

5. Watanabe M, Sugawara T, Hiramatsu A, Ohira T, Ogata K, Yamamoto S: **Relationships Between Macular Function and the Nei Vfq-25 in Patients With Retinitis Pigmentosa.** *Investigative Ophthalmology & Visual Science* 2008, **49:**2180-2180 & 1552-5783.

6. Siqueira RC, Messias A, Messias K, Arcieri RS, Ruiz MA, Souza NF, Martins LC, Jorge R: **Quality of life in patients with retinitis pigmentosa submitted to intravitreal use of bone marrow-derived stem cells (Reticell-clinical trial).** *Stem cell research & therapy* 2015, **6:**29 & 1757-6512.

7. Burstedt MS, Monestam E: **Self-reported quality of life in patients with retinitis pigmentosa and maculopathy of Bothnia type.** *Clinical Ophthalmology* 2010, **4:**147-154.

8. Burstedt MS, Monestam E, Sandgren O: **Associations between specific measures of vision and vision-related quality of life in patients with bothnia dystrophy, a defined type of retinitis pigmentosa.** *Retina* 2005, **25:**317-323.

9. Menzel-Severing J, Laube T, Brockmann C, Bornfeld N, Mokwa W, Mazinani B, Walter P, Roessler G: **Implantation and explantation of an active epiretinal visual prosthesis: 2-year follow-up data from the EPIRET3 prospective clinical trial.** *Eye* 2012, **26:**501-509 & 1476-5454.

10. Thurston M, Thurston A, McLeod J: **Socio-emotional effects of the transition from sight to blindness.** *British Journal of Visual Impairment* 2010, **28:**90-112 & 0264-6196.

11. Levinson JD, Joseph E, Ward LA, Nocera JR, Pardue MT, Bruce BB, Yan J: **Physical Activity and Quality of Life in Retinitis Pigmentosa.** *Journal of Ophthalmology* 2017, **2017:**6950642.

12. Ivanov IV, Mackeben M, Vollmer A, Martus P, Nguyen NX, Trauzettel-Klosinski S: **Eye movement training and suggested gaze strategies in tunnel vision-a randomized and controlled pilot study.** *PLoS One* 2016, **11**.

13. Azoulay L, Chaumet-Riffaud P, Jaron S, Roux C, Sancho S, Berdugo N, Audo I, Sahel JA, Mohand-Said S: **Threshold levels of visual field and acuity loss related to significant decreases in the quality of life and emotional states of patients with retinitis pigmentosa.** *Ophthalmic Research* 2015, **54:**78-84.

14. Chaumet-Riffaud AE, Chaumet-Riffaud P, Cariou A, Devisme C, Audo I, Sahel JA, Mohand-Said S: **Impact of Retinitis Pigmentosa on Quality of Life, Mental Health, and Employment Among Young Adults.** *American Journal of Ophthalmology* 2017, **177:**169-174.

15. Sainohira M, Yamashita T, Terasaki H, Sonoda S, Miyata K, Murakami Y, Ikeda Y, Morimoto T, Endo T, Fujikado T, et al: **Quantitative analyses of factors related to anxiety and depression in patients with retinitis pigmentosa.** *PLoS One* 2018, **13:**e0195983.

16. Fenwick EK, O'Hare F, Deverell L, Ayton LN, Luu CD, McSweeney S, Bentley SA, Guymer RH, Finger RP: **Rasch Analysis of the Independent Mobility Questionnaire.** *Optometry and Vision Science* 2016, **93:**181-187.

17. Berson EL, Rosner B, Sandberg MA, Weigel-DiFranco C, Moser A, Brockhurst RJ, Hayes KC, Johnson CA, Anderson EJ, Gaudio AR, et al: **Further evaluation of docosahexaenoic acid in patients with retinitis pigmentosa receiving vitamin A treatment: subgroup analyses.** *Archives of Ophthalmology* 2004, **122:**1306-1314.

18. Berson EL, Rosner B, Sandberg MA, Weigel-DiFranco C, Moser A, Brockhurst RJ, Hayes KC, Johnson CA, Anderson EJ, Gaudio AR, et al: **Clinical trial of docosahexaenoic acid in patients with retinitis pigmentosa receiving vitamin A treatment.** *Archives of Ophthalmology* 2004, **122:**1297-1305.

19. Woods RL, Giorgi RG, Berson EL, Peli E: **Extended wearing trial of Trifield lens device for 'tunnel vision'.** *Ophthalmic & Physiological Optics* 2010, **30:**240-252.

20. Yamamoto S, Sugawara T, Murakami A, Nakazawa M, Nao-i N, Machida S, Wada Y, Mashima Y, Myake Y: **Topical isopropyl unoprostone for retinitis pigmentosa: microperimetric results of the phase 2 clinical study.** *Ophthalmology and therapy* 2012, **1:**5 & 2193-8245.

21. Anil K, Garip G: **Coping strategies, vision-related quality of life, and emotional health in managing retinitis pigmentosa: a survey study.** *BMC Ophthalmology* 2018, **18:**21.

22. Mancil RM, Mancil GL, King E, Legault C, Munday J, Alfieri S, Nowakowski R, Blasch BB: **Improving nighttime mobility in persons with night blindness caused by retinitis pigmentosa: A comparison of two low-vision mobility devices.** *Journal of Rehabilitation Research and Development* 2005, **42:**471-486.

23. Burstedt MS, Mönestam E: **Self-reported quality of life in patients with retinitis pigmentosa and maculopathy of Bothnia type.** *Clinical Ophthalmology* 2010, **4:**147.

24. Burstedt MSI, Mönestam E, Sandgren O: **Associations between specific measures of vision and vision-related quality of life in patients with bothnia dystrophy, a defined type of retinitis pigmentosa.** *Retina* 2005, **25:**317-323 & 0275-0004X.

25. Sugawara T, Hagiwara A, Hiramatsu A, Ogata K, Mitamura Y, Yamamoto S: **Relationship between peripheral visual field loss and vision-related quality of life in patients with retinitis pigmentosa.** *Eye* 2010, **24**.

26. Siqueira RC, Messias A, Messias K, Arcieri RS, Ruiz MA, Souza NF, Martins LC, Jorge R: **Quality of life in patients with retinitis pigmentosa submitted to intravitreal use of bone marrow-derived stem cells (Reticell -clinical trial).** *Stem Cell Research & Therapy* 2015, **6:**29.

27. Pesudovs K, Gothwal VK, Wright T, Lamoureux EL: **Remediating serious flaws in the National Eye Institute Visual Function Questionnaire.** *Journal of Cataract and Refractive Surgery* 2010, **36:**718-732.

28. **Rasch G. Probabilistic Models for Some Intelligence and Attainment Tests. .** *Danmarks Paedagogiske Institute* 1960, **Copenhagen, Denmark**.

29. Guttman L: *The basis for scalogram analysis.* Bobbs-Merrill, College Division; 1974.

30. Smith RM: **Fit analysis in latent trait measurement models.** *Journal of applied measurement 1090-655X* 2000.

31. Ludlow LH, Haley SM: **Rasch model logits: Interpretation, use, and transformation.** *Educational and Psychological Measurement* 1995, **55:**967-975 & 0013-1644.

32. Linacre JM: **Misfit statistics for rating scale categories.** *Rasch Measurement Transactions* 1995, **9:**450.

33. Linacre JM: **Detecting multidimensionality: which residual data-type works best?** *Journal of Outcome Measurement* 1998, **2:**266-283.

34. Bond TG, Fox CM: *Applying the Rasch Model: Fundamental Measurement in the Human Sciences.* 3rd edn. New York, NY: Routledge; 2015.

35. **Reliability and separation of measures** [<http://www.winsteps.com/winman/reliability.htm>]

36. Wright BD: **Solving measurement problems with the Rasch model.** *Journal of Educational Measurement* 1977, **14:**97-116.

37. Linacre JM: **Winsteps® Rasch measurement computer program.** *Beaverton, Oregon* 2019, **Winsteps.com**.

38. Marella M, Pesudovs K, Keeffe JE, O'Connor PM, Rees G, Lamoureux EL: **The psychometric validity of the NEI VFQ-25 for use in a low-vision population.** *Investigative Ophthalmology & Visual Science* 2010, **51:**2878-2884.

39. Pesudovs K, Burr JM, Harley C, Elliott DB: **The development, assessment, and selection of questionnaires.** *Optometry and Vision Science* 2007, **84:**663-674.

40. Garamendi E, Pesudovs K, Stevens MJ, Elliott DB: **The Refractive Status and Vision Profile: Evaluation of psychometric properties and comparison of Rasch and summated Likert-scaling.** *Vision Research* 2006, **46:**1375-1383.

41. Gothwal VK, Wright TA, Lamoureux EL, Pesudovs K: **Measuring outcomes of cataract surgery using the Visual Function Index-14.** *Journal of Cataract and Refractive Surgery* 2010, **36:**1181-1188.

42. Massof RW: **The measurement of vision disability.** *Optometry & Vision Science* 2002, **79:**516-552.
